# Ligand-Based Virtual Screening and Molecular Docking of Benzimidazoles as Potential Inhibitors of Triosephosphate Isomerase Identified New Trypanocidal Agents

**DOI:** 10.3390/ijms231710047

**Published:** 2022-09-02

**Authors:** Lenci K. Vázquez-Jiménez, Alfredo Juárez-Saldivar, Rogelio Gómez-Escobedo, Timoteo Delgado-Maldonado, Domingo Méndez-Álvarez, Isidro Palos, Debasish Bandyopadhyay, Carlos Gaona-Lopez, Eyra Ortiz-Pérez, Benjamín Nogueda-Torres, Esther Ramírez-Moreno, Gildardo Rivera

**Affiliations:** 1 Laboratorio de Biotecnología Farmacéutica, Centro de Biotecnología Genómica, Instituto Politécnico Nacional, Reynosa 88710, Mexico; 2 Escuela Nacional de Ciencias Biológicas, Instituto Politécnico Nacional, Ciudad de México 11340, Mexico; 3 Unidad Académica Multidisciplinaria Reynosa-Rodhe, Universidad Autónoma de Tamaulipas, Reynosa 88779, Mexico; 4 Department of Chemistry and SEEMS, University of Texas Rio Grande Valley, Edinburg, TX 78539, USA; 5 Escuela Nacional de Medicina y Homeopatía, Instituto Politécnico Nacional, Ciudad de México 07320, Mexico

**Keywords:** molecular dynamics, molecular docking, inhibitors, triosephosphate isomerase, *Trypanosoma cruzi*

## Abstract

*Trypanosoma cruzi* (*T. cruzi*) is a parasite that affects humans and other mammals. *T. cruzi* depends on glycolysis as a source of adenosine triphosphate (ATP) supply, and triosephosphate isomerase (TIM) plays a key role in this metabolic pathway. This enzyme is an attractive target for the design of new trypanocidal drugs. In this study, a ligand-based virtual screening (LBVS) from the ZINC15 database using benzimidazole as a scaffold was accomplished. Later, a molecular docking on the interface of *T. cruzi* TIM (*Tc*TIM) was performed and the compounds were grouped by interaction profiles. Subsequently, a selection of compounds was made based on cost and availability for in vitro evaluation against blood trypomastigotes. Finally, the compounds were analyzed by molecular dynamics simulation, and physicochemical and pharmacokinetic properties were determined using SwissADME software. A total of 1604 molecules were obtained as potential *Tc*TIM inhibitors. BP2 and BP5 showed trypanocidal activity with half-maximal lytic concentration (LC_50_) values of 155.86 and 226.30 µM, respectively. Molecular docking and molecular dynamics simulation analyzes showed a favorable docking score of BP5 compound on *Tc*TIM. Additionally, BP5 showed a low docking score (−5.9 Kcal/mol) on human TIM compared to the control ligand (−7.2 Kcal/mol). Both compounds BP2 and BP5 showed good physicochemical and pharmacokinetic properties as new anti-*T. cruzi* agents.

## 1. Introduction

Chagas disease or American trypanosomiasis is a “neglected tropical” disease because it occurs mainly in low-income countries [1]. This disease causes 56,000 new cases annually in Latin America, and it is estimated that it causes approximately 12,000 deaths per year [2]. Nifurtimox (Nfx) and benznidazole (Bzn) are the only drugs available on the market for the treatment of this disease, which are not suitable because they cause severe adverse effects. Additionally, both drugs have poor efficacy in the chronic phase [3].

Chagas disease is caused by the parasite *Trypanosoma cruzi* (*T. cruzi*), a protozoan dependent on glycolysis as the only source of supply of adenosine triphosphate (ATP) for energy generation [4]. Therefore, glycolytic enzymes are potential targets in the search for new therapeutic agents [5]. In this context, triosephosphate isomerase (TIM) enzyme has been proposed by different research groups as a target for the design of new drugs against *T. cruzi* [6,7]. 

TIM from *T. cruzi* (*Tc*TIM) catalyzes the reversible interconversion of the products that come from the catalysis of aldolase (glyceraldehyde-3-phosphate and dihydroxyacetone phosphate) through an intermediate enediol (alkenol), in the fifth step of the glycolysis pathway [8]. *Tc*TIM structurally adopts an α/ß barrel folding, where each TIM monomer is made up of eight parallel ß strands that form the inner part of the barrel and are surrounded by eight α helices that are joined by handles [9]. An important site for the binding of various small molecules is the interface [5,10,11,12,13,14]; it is found among the monomers and occupies a significant part of the molecular surface area of each monomer (1490 Å^2^) in addition to being mainly composed of loops 1, 2, 3, 4 and 8 [15,16,17].

In the search for new molecules with biological activity, benzimidazole derivatives have presented a variety of activities [18,19,20] highlighting the antiparasitic activity against *T. cruzi* [21,22]. Among these compounds, two 2,3-dihydroimidazo[1,2-a]benzimidazole analogs have been shown to have trypanocidal effects (IC_50_ = 1.10 and 2.10 μM), with a better activity than the reference drug Bzn (IC_50_ = 20.7 μM) and without severe cytotoxicity against U2OS cells (CC_50_ = 36.5 and 18.8 μM) [22]. Other authors reported that benzimidazole derivatives (IC_50_ = 5 μM) are more active than Bzn (IC_50_ = 7.5 μM) to inhibit the growth of the parasite in epimastigote form [23]. Furthermore, the benzimidazole scaffold is considered a privileged structure in medicinal chemistry, as it has the potential to interact with different biological targets, including *Tc*TIM [24,25]. 

In the present work, a ligand-based virtual screening (LBVS) from the ZINC15 database using benzimidazole substructure was carried out; the compounds obtained were analyzed by molecular docking, and subsequently, a selection of compounds was made based on the interaction profile and docking score for an in vitro evaluation against trypomastigotes of *T. cruzi* and a molecular dynamics simulation analysis. Benzimidazole derivatives have been used in drug designing; however, these kinds of compounds have shown some disadvantages such as hepatotoxicity, among others [26,27,28,29,30]. Therefore, in this study, a prediction of the adsorption, distribution, metabolism and excretion (ADME) properties by computational analysis was performed. Finally, to know their potential selectivity, a molecular docking analysis and molecular dynamics simulation of the compounds against human TIM (*Hs*TIM) were performed.

## 2. Results

### 2.1. TcTIM Inhibitors Analysis

In a first step, ten compounds with inhibitory activity against *Tc*TIM were analyzed by molecular docking. Table 1 shows the docking score and interactions profile at the *Tc*TIM interface. Six compounds are benzimidazole derivatives (L1–L6), and four presented a different structure. L7 and 3-(2-benzothiazolylthio-1-propanesulfonic acid (BTS) are benzothiazoles; L8, a thiazole; and L9, a cyclohexanone derivative. The control ligands derived from benzimidazole (L1-L6) show inhibition percentages of 48% to 69%, at a concentration of 200 µM (except the ligand L3) [21,25]. Ligands L7-L9 show IC_50_ values between 0.086 and 8.0 µM (Table 1) [30,31,32,33].

**Figure 1:**
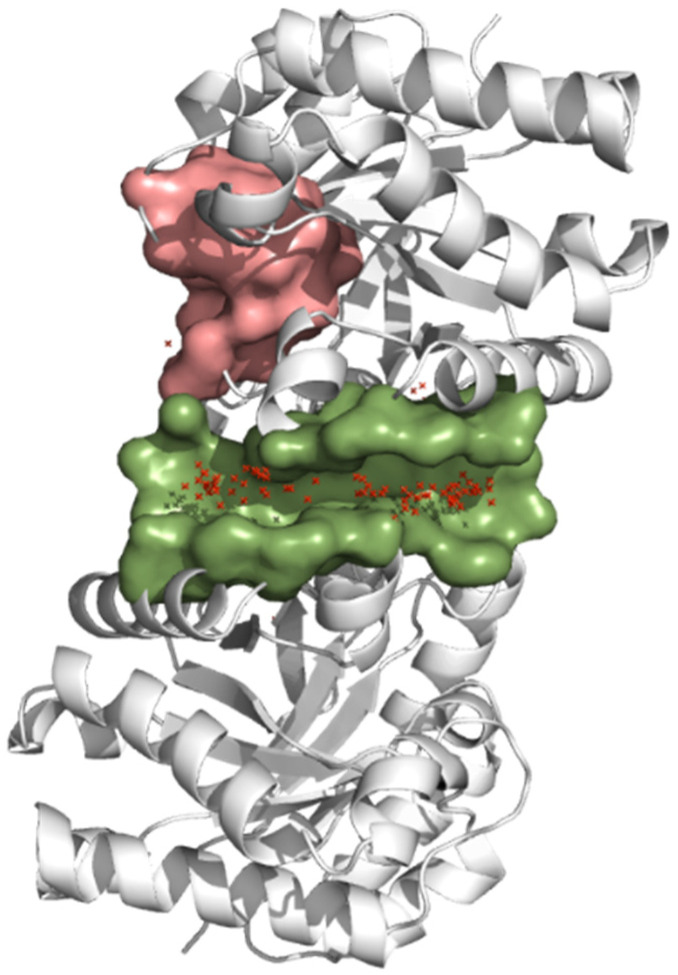
Binding site prediction by the DoGSiteScorer web Server and blind molecular docking in *Tc*TIM. The protein is represented in gray, the interface is represented in green, and the ligands in red crosses.

To determine the most favorable binding site of control ligands for molecular docking analysis, the structure of *Tc*TIM protein (PDB 1SUX) was analyzed with the DoGSiteScorer tool. The results showed two possible binding sites (interface and the active site) with a score (drug score) greater than 0.5. This value indicates that both sites could bind with high affinity to a drug. Additionally, blind molecular docking was performed (PyRx software was used), which showed that most of the control ligands bind at the interface, with docking scores ranging from −6.2 to −8.9 Kcal/mol (Table 1). The results of both analyses showed that the best binding site for control ligands is the interface (Figure 1).

### 2.2. Molecular Docking of Control Ligands at the TcTIM Interface

Once the possible binding site of the control ligands was identified, a molecular docking analysis was carried out at the *Tc*TIM interface (PyRx software was used). Table 1 shows the docking scores of each of the control ligands evaluated, as well as the interactions with the amino acid residues. The compound with the lowest score (−8.9 Kcal/mol) was ligand L1. The compound BTS co-crystallized presented the highest docking score of −6.2 Kcal/mol. 

### 2.3. LBVS from ZINC15 Database and Molecular Docking Analysis

Considering the benzimidazole scaffold, a LBVS of 750 million compounds from ZINC15 database was carried out. A total of 67,141 molecules per substructure was obtained. Subsequently, the Lipinski’s rule of five was applied as an inclusion criterion, obtaining 53,410 molecules. These compounds were analyzed by molecular docking at the *Tc*TIM interface, obtaining 1604 compounds that showed a binding energy value between −10.6 and −8.9 Kcal/mol. Subsequently, these compounds were grouped based on the type of interactions present in each compound (using the scikit-learn library and the DataWarrior program), obtaining ten groups (Table 2) [32,33]. Group three had the largest number of compounds (206), and group six had the fewest number of compounds (103).

**Figure 2:**
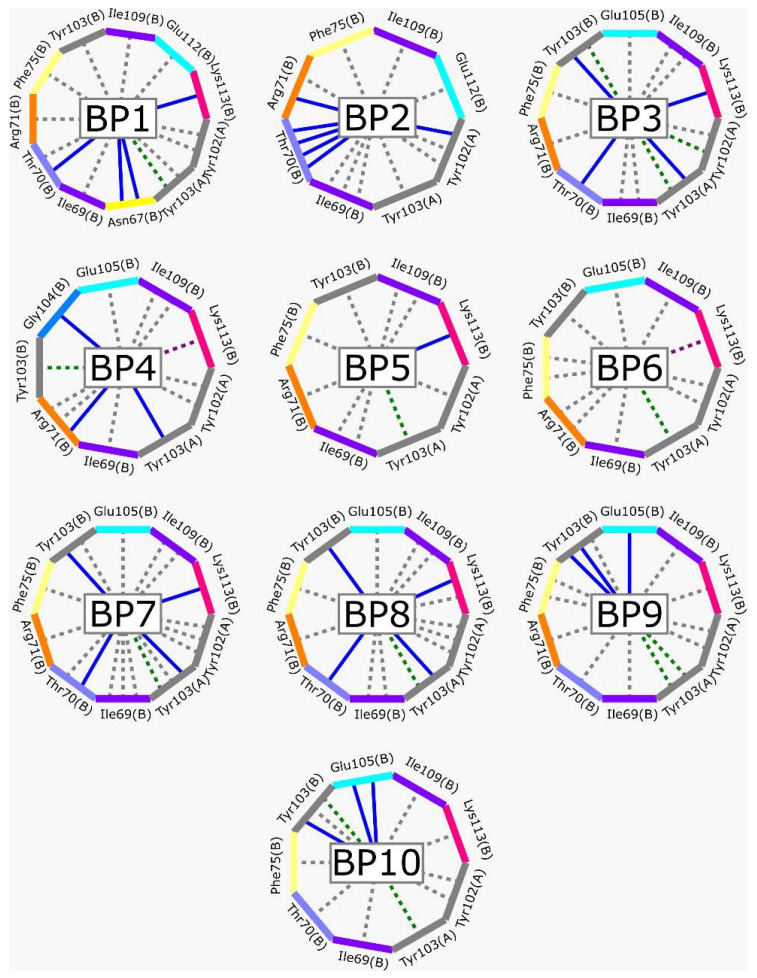
Lead compound of each group obtained by interaction profile analysis. Hydrophobic interactions are shown in gray, hydrogen bonds in blue, π-stacking in green, and cation-π interactions in purple.

Figure 2 shows the interactions of the lead compound of each group. The interactions that predominated in the ten lead compounds were hydrophobic interactions, hydrogen bonds, and π-stacking. The most common interaction is hydrophobic with amino acid residues Tyr102 (A), Ile109 (B), and Ile69 (B) in all ten compounds, followed by hydrogen bonding interactions with amino acid residues Thr70 (B), Tyr103 (B), and Lys113 (B) in six compounds. 

### 2.4. Trypanocidal Activity

Based on availability and accessibility, compounds BP2 and BP5 (Table 2) were purchased to be evaluated against blood trypomastigotes of the NINOA and INC-5 strains of *T. cruzi* to determine their trypanocidal activity. The results are shown in Table 3. Compound BP2 presented better half-maximal lytic concentration (LC_50_) against the NINOA strain (LC_50_ = 155.86 ± 3.4 µM), a similar value to Bzn, however, two times less than Nfx. Compound BP5 showed similar activity against the two strains evaluated (LC_50_ = 179.55 ± 19.7 and 179.71 ± 19.0 µM, respectively). 

Compounds BP2 and BP5 showed trypanocidal activity and were considered as potential *Tc*TIM inhibitors due to their high docking scores (−10.4 and −10.2 Kcal/mol, respectively); therefore, a molecular dynamic simulation at the *Tc*TIM interface was done to predict the stability of ligand–*Tc*TIM complex.

### 2.5. Molecular Dynamics Simulation

**Figure 3:**
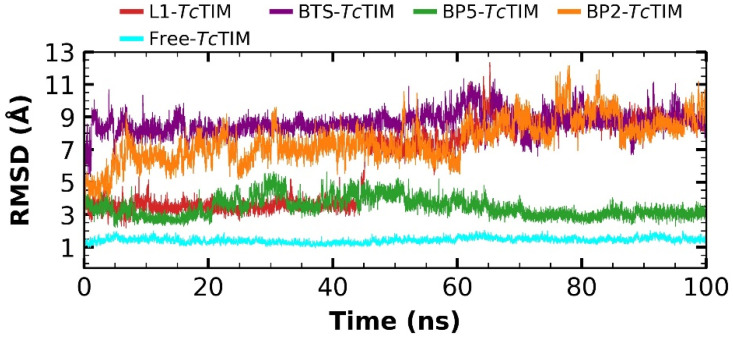
RMSD of molecular dynamics simulation of free *Tc*TIM, benzimidazole derivatives, and the BTS ligand in complex with *Tc*TIM.

In the molecular dynamics analysis, the *Tc*TIM (free) showed a Root Mean Square Deviation (RMSD) from 0.33 to 2.04 Å, with a difference in oscillation of 1.71 Å and a mean of 1.43 Å (Figure 3). The L1–*Tc*TIM complex showed stability in the first 45 ns, with an RMSD of 2.17 Å and a mean of 6.17 Å. Overall, BP2–*Tc*TIM complex showed a fluctuation from 0.66 to 12.16 Å, with a difference between the oscillations of 11.5 Å, and a mean of 7.55 Å. Meanwhile, the BP5–*Tc*TIM complex showed similar stability as L1 in the first 45 ns. Additionally, in the next 55 ns, it showed good stability without big fluctuations (RMSD from 0.97 Å to 6.07 Å, showing a difference between the oscillations of 5.11 Å and a mean of 3.59 Å). Additionally, the RMSD of the ligand BTS co-crystallized with the protein was analyzed, which presented an RMSD value of 1.02 to 11.36 Å, and the difference in the oscillation is 10.34 Å, with a mean of 8.65 A.

**Figure 4:**
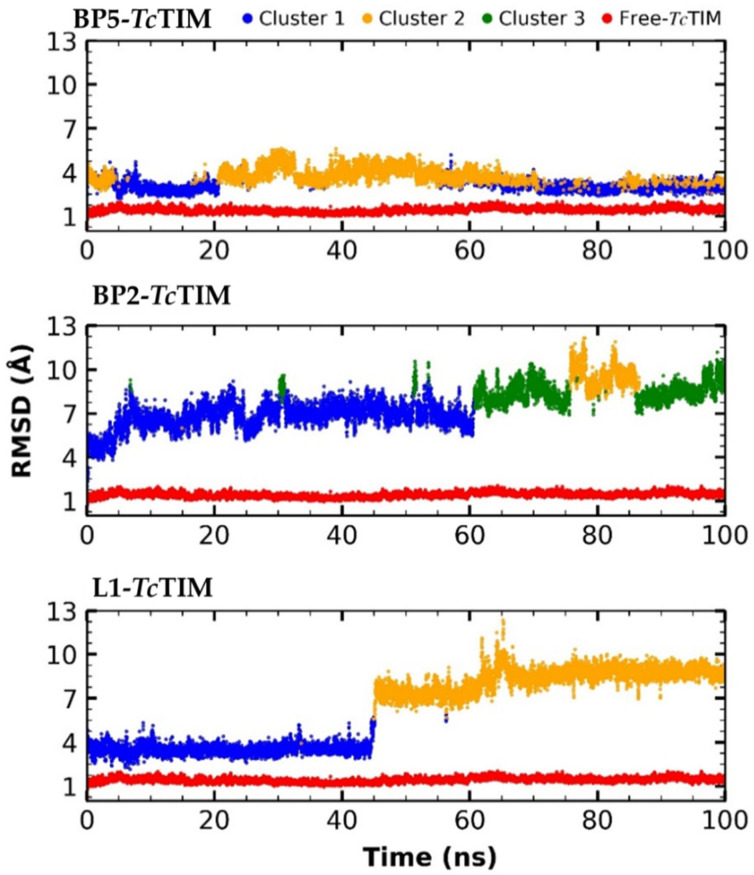
RMSD clustering of molecular dynamics simulation of free *Tc*TIM and benzimidazole derivatives in complex with *Tc*TIM.

**Figure A1:**
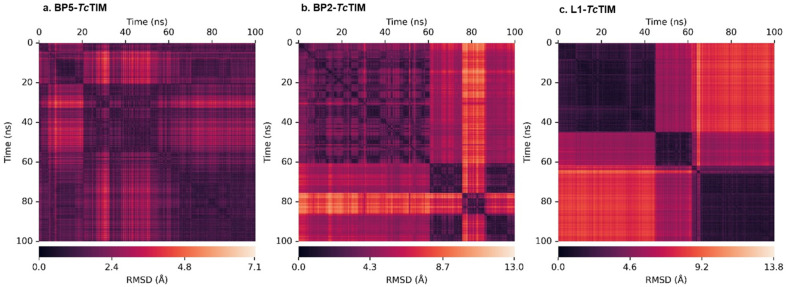
Matrices for RMSD cluster analysis.

**Figure A2:**
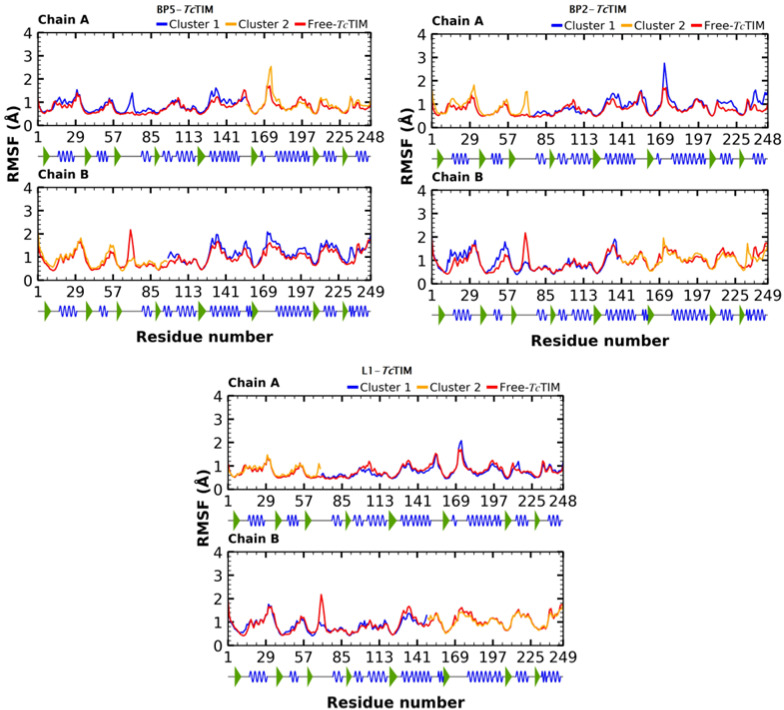
RMSF cluster analysis.

**Figure A3:**
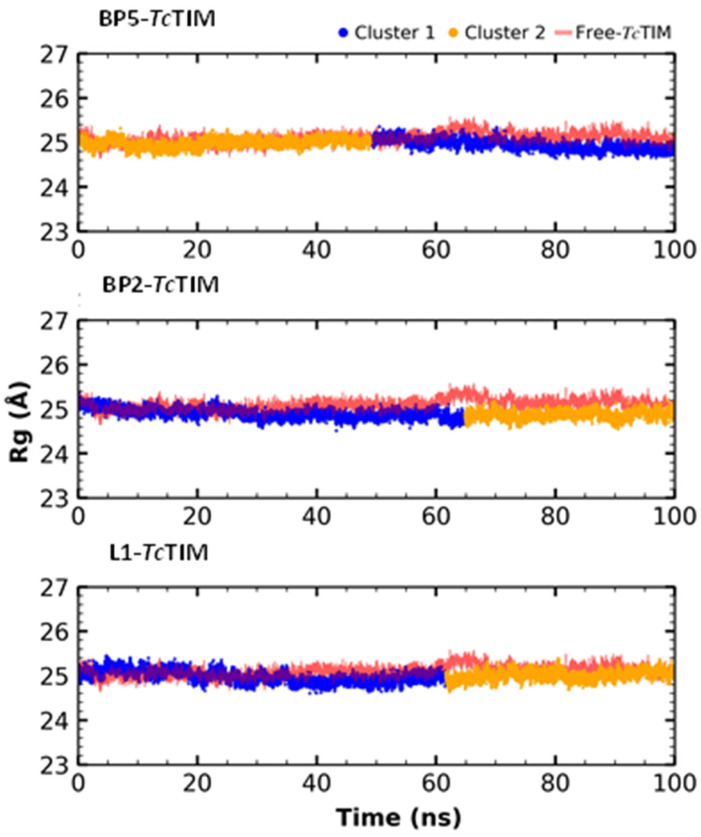
Rg cluster analysis.

**Figure A4:**
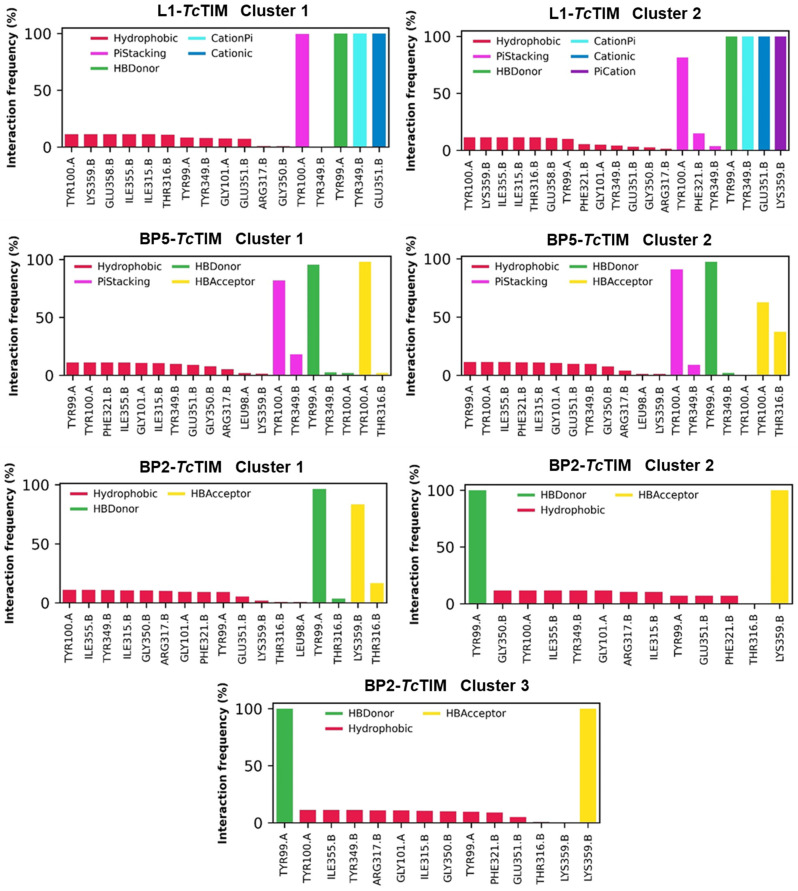
Frequency of interactions present in each cluster by RSMD.

The RMSD obtained could be influenced by changes that occur in other parts of the structure; therefore, a clustering of the RMSD of each ligand-protein versus the simulation time was performed (Figure 4). An RMSD matrix of each complex was made (Appendix A, Figure A1), and the Ward method and the Euclidean clustering distance were applied to obtain more information on the trajectory of molecular dynamics [34,35]. For the RMSD clustering of BP2, three clusters were observed (corresponding to 59.5, 10.4 and 30.1% frames, respectively), whereas for BP5 and L1, two clusters were observed (for BP5 of 51 and 49 % frames and L1 of 45.1 and 54.8%, respectively) [36]. Additionally, the clustering of the RMSF and Rg was formed (Appendix A, Figure A2, and Figure A3, respectively). In addition to the clustering of the RMSD, an interaction frequency analysis by group was performed to observe the rate of interactions present throughout the analyzed time, which is shown in Appendix A (Figure A4). 

**Figure 5:**
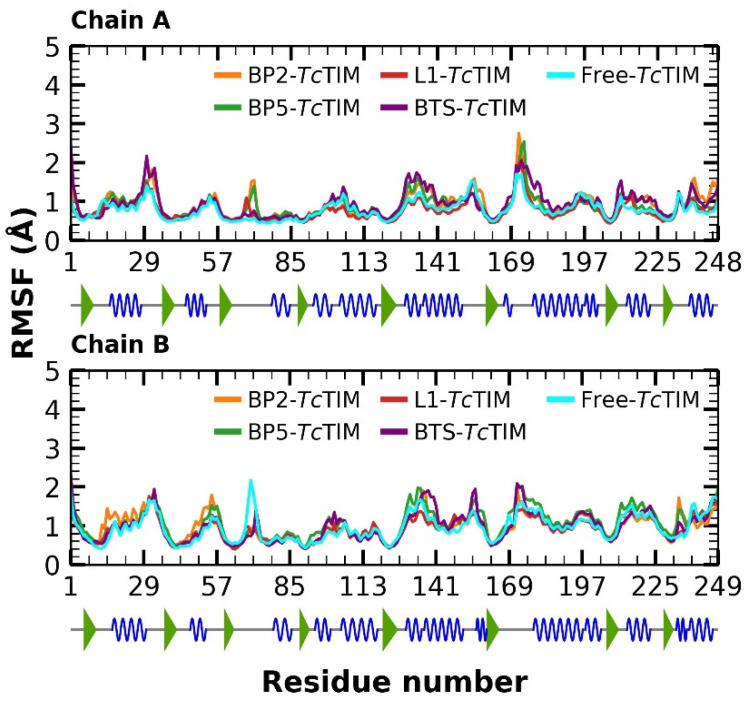
RMSF of molecular dynamics simulation of free *Tc*TIM, benzimidazole derivatives, and the BTS ligand in complex with *Tc*TIM.

The Root Mean Square Fluctuation (RMSF) was also analyzed during molecular dynamics simulation (Figure 5). The RMSF showed a very similar fluctuation pattern between the free protein and the protein in complex with the ligands, especially with the BP5 compound. However, the RMSF calculation revealed that the complex with the BP2 ligand showed a high fluctuation in some regions according to the RMSD pattern as well as in the L1 and BTS ligands in variable proportions.

**Figure 6:**
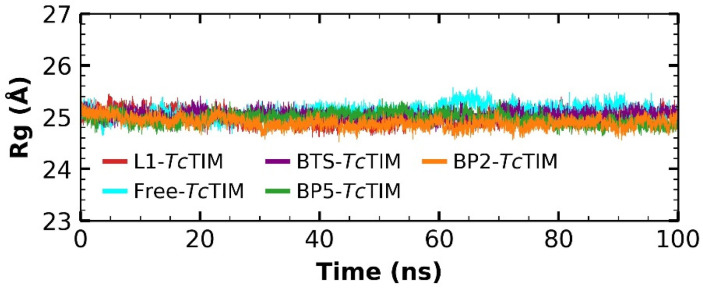
Rg of molecular dynamics simulation of free *Tc*TIM, benzimidazole derivatives and the BTS ligand in complex with *Tc*TIM.

Additionally, the radius of gyration (Rg) of the free protein was analyzed, as well as of the protein in complex with the ligands (L1, BP2, BP5 and BTS). This parameter allows the calculation of the structural variations of the protein during the analysis of the molecular dynamics [37]. The free *Tc*TIM folding maintains an almost constant fluctuation between 24.64 and 25.57 Å, with a difference in the oscillation of 0.93 Å over 100 ns (Figure 6). The L1–*Tc*TIM complex presented fluctuations between 24.60 and 25.44 Å, with a difference of 0.83 Å. The BP2–*Tc*TIM complex showed a fluctuation from 24.51 to 25.36 Å, with a difference of 0.84 Å, whereas the complex with the BP5 compound fluctuated from 24.72 to 25.50 Å, with a difference of 0.77 Å, this being the one with the smallest difference of the three complexes. On the other hand, the BTS–*Tc*TIM complex showed a fluctuation with a minimum of 24.68 Å and a maximum of 25.40 Å, whereas the difference in oscillation was 0.72 Å.

Additionally, the mean run values of the different components of the calculated Molecular Mechanics Poisson–Boltzmann Surface Area (MMGBSA) binding free energies were determined (Table 4).

### 2.6. Analysis of Molecular Physicochemical Properties

Molecular physicochemical properties were calculated for the compounds BP2 and BP5 (SwissADME website was used). The descriptors obtained (Table 5) were: physicochemical properties (number of hydrogen bond acceptors, number of hydrogen bond donors, number of rotational bonds, polar surface area, molecular weight, and partition coefficient), solubility coefficient, pharmacokinetics (human gastrointestinal absorption, blood-brain permeability, P-glycoprotein substrate, and inhibition of different CYP450 isoforms), and hepatotoxicity (ProTox-II server was used).

### 2.7. Molecular Docking at the HsTIM Interface

**Figure 7:**
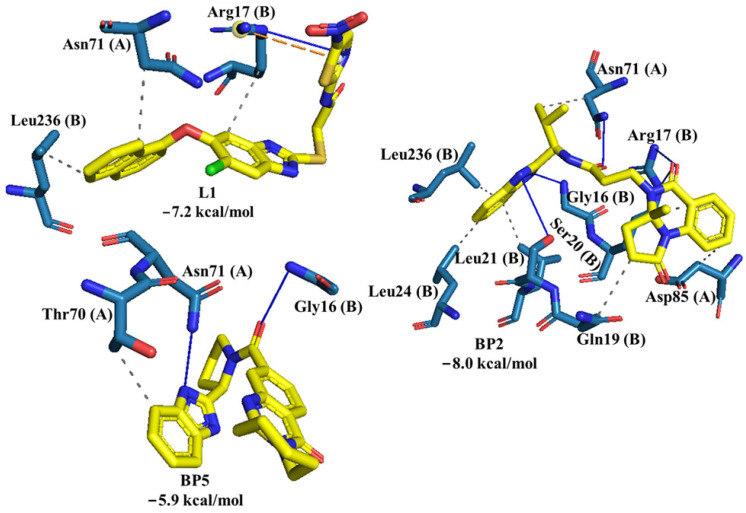
Interaction profile of L1, BP2, and BP5 at the *Hs*TIM interface. Hydrogen bonds are shown as blue lines and hydrophobic interaction as gray dashed lines. Yellow dashed lines represent Salt Bridge interaction. Interactions were obtained using the PLIP online server.

*Hs*TIM and *Tc*TIM interfaces have been described with 52% sequence identity [38]. Therefore, compounds capable of specifically disrupting the *Tc*TIM interface but not the *Hs*TIM interface would be expected to be converted to selective trypanocidal compounds. Thus, the binding affinity of BP2, BP5, and the control ligand (L1) at the *Hs*TIM interface was analyzed by molecular docking (Figure 7). L1 presented a docking score of −7.2 Kcal/mol, BP2 presented a score of −8.0 Kcal/mol, and BP5 presented a docking score of −5.9 Kcal/mol.

### 2.8. Molecular Dynamics Simulation at the HsTIM Interface

**Figure 8:**
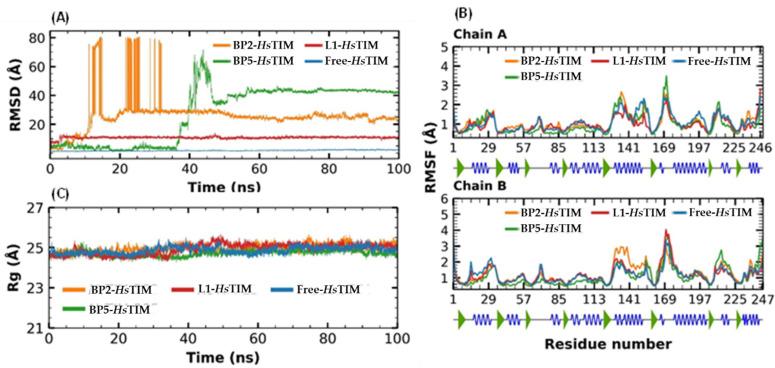
Analysis of molecular dynamics simulations of L1–, BP2– and BP5–*Hs*TIM complex. (**A**) RMSD diagram of the three complexes. (**B**) RMSF diagram of the three complexes. (**C**) Rg diagram of the three complexes.

To complement the results obtained in the molecular docking analysis of L1, BP2, and BP5 at the *Hs*TIM interface, molecular dynamics simulations were carried out (Figure 8). The RMSD values of *Hs*TIM (free) remained constant with a minimum fluctuation of 0.3 Å and a maximum of 3.2 Å, and a difference in oscillation of 2.9 Å and a mean of 1.87 Å (Figure 8A). The RMSD value of L1–*Hs*TIM reached its equilibrium at 3 ns with a minimum fluctuation of 0.3 to a maximum fluctuation of 3.2 Å, with a difference in oscillation of 2.9 Å and a mean of 1.87 Å. Subsequently, the RMSD values for the BP2 and BP5 complexes showed a rise in RMSD values, with a mean fluctuation of 24.69 and 27.91 Å, respectively. Figure 8B shows the RMSF plot, which showed high fluctuations in most of the regions according to the RMSD pattern. 

In addition, the Rg was determined (Figure 8C) for the protein alone (24.36 to 25.42 Å, difference is 1.06 Å and a mean of 24.89 Å) as well as for the complexes. For L1 and BP2 complex, the Rg was very similar with differences in the oscillation of 1.36 and 1.30 Å, and with means of 24.95 and 24.99 Å, respectively. BP5–*Hs*TIM presented a minimum fluctuation of 24.26 Å and a maximum of 25.29 Å, with a difference of 1.03 Å and a mean of 24.71 Å.

Finally, as for the Ligand–*Tc*TIM complexes, the mean execution values of the different components of the free binding energies of the MMGBSA of the molecular mechanics calculated for the Ligand–*Hs*TIM complexes were also determined, which are shown in Table 4.

## 3. Discussion

### 3.1. TcTIM Inhibitors Analysis

Ten compounds reported in the literature with inhibitory activity against *Tc*TIM were selected as control ligands for molecular docking analysis. Compound L1 showed the lowest score (−8.9 Kcal/mol) [22]; this benzimidazole derivative has a 5-nitro-1,3-thiazol-2-yl aminocarbonyl-2-methylsulfanyl fragment at 2-position, and a 1-naphthyloxy group and a chlorine atom at 5- and 6-position, respectively. L4 (−7.3 Kcal/mol), L5 (−7.2 Kcal/mol), and L6 (−7.2 Kcal/mol) have a similar structure as L1. However, the 1-naphthyloxy group was replaced by a methoxycarbonyl group. This replacement could explain the difference in the docking values between these compounds, according to the interactions calculated. L1 has a larger structure that generates a greater number of hydrophobic interactions. Compounds L2, L3, L7 (1,2,4-thiadiazole derivatives), and L8 (benzothiazole derivative) presented higher docking scores (between −6.8 and −6.9 Kcal/mol). On the other hand, the compound BTS co-crystallized with the protein at the interface binding site [39] presented the highest docking score of −6.2 Kcal/mol. These results suggest that the benzimidazole scaffold is key to obtaining a better binding at the *Tc*TIM interface.

All control ligands showed hydrophobic interactions, hydrogen bonds, and π-stacking interactions. Hydrophobic interactions have been reported to have a greater effect on the docking score because this type of ligand-protein interaction is important in the function of a molecule [40]. All control ligands showed interactions with the amino acid residues Tyr102 (A), Tyr103 (A), and Ile69 (B), suggesting a triad catalytic. Various researchers have described interactions with residues important in the binding of compounds at the *Tc*TIM interface. Other interactions with Asn67, Phe75, Thr70, Glu105, and Lys113 have been reported [31,41,42,43].

### 3.2. LBVS from ZINC15 Database and Molecular Docking Analysis

The LBVS allowed 67,141 molecules with a benzimidazole scaffold to be obtained from the ZINC15 database. After that, 1604 molecules by molecular docking showed better binding energy than ligand L1. The lead compound of the ten groups obtained from molecular docking analysis showed interactions with amino acid residues previously reported as important for the binding on the *Tc*TIM interface, such as Tyr102 (A), Tyr103 (A), and Ile69 (B) [25,26,29,31]. The presence of hydrogen bonds, as well as hydrophobic interactions, also promote the formation of stronger and more robust ligand–protein complexes [32]. There were also some π-stacking interactions that are also related to binding stability [6], these being with the amino acid residues Tyr103 (A), Tyr102 (A), and Tyr103 (B) in all compounds except compound BP2. 

On the other hand, only compounds BP4 and BP6 presented a cation-π interaction with Lys113 (B), whereas compound BP1 additionally had interactions with residues Glu115 (B) and Asn67 (B), which contributed to stability through hydrophobic interactions [43]. It is worth mentioning that the control ligands also presented these types of interactions with most of the residues, where Tyr102 (A), Tyr103 (A), and Ile69 (B) stand out in the ten control ligands.

### 3.3. Trypanocidal Activity

According to low price and accessibility, compounds BP2 and BP5 were purchased for trypanocidal effect analysis. Both compounds showed trypanocidal activity against the trypomastigote form of *T. cruzi*, the infective form of the parasite in the mammalian host [44].

### 3.4. Molecular Dynamics Simulation at the TcTIM Interface

The free protein (*Tc*TIM), L1-*Tc*TIM, B2-*Tc*TIM, B5-*Tc*TIM, and BTS-*Tc*TIM were analyzed by molecular dynamics simulations to determine the stability of the complex formed [44,45]. In addition, the complex of the ligand co-crystallized with the protein (BTS-*Tc*TIM) was analyzed to expand the information that has been described in the literature.

L1-*Tc*TIM showed a fluctuation peak in RMSD values, suggesting a change in the initial binding position, outside of the interface [43]. According to the literature, high RMSD values of a complex compared to the initial framework indicate a change in the initial binding position [33,40]. The results obtained suggest that BP5–*Tc*TIM is better than the control ligand (L1–*Tc*TIM) and the co-crystallized ligand (BTS–*Tc*TIM), since it has been described that the complex with the lowest RMSD and with minimal differences between the oscillations is the most stable complex [46,47]. 

Additionally, RMSD clustering was carried out (L1–*Tc*TIM, B2–*Tc*TIM and B5–*Tc*TIM). A distribution by groups was clearly observed. Notably, two of them could be dynamic events, whereas the third group is best described as a conformational transition from one group to another (Figure 4) [36].

In addition, the frequencies of the interactions by group (see Appendix A) show that L1 is the compound that presented the highest number of different interactions in both groups, highlighting π-stacking interactions with Tyr100 (A) (99.75 and 81.58% presence over time in each group, respectively) and donors of hydrogen bonds with Tyr99 (A), among others such as cation-π and cationic interactions that remain for approximately 100% of the simulation time (Figure A2). In the case of BP5, the interaction frequency also shows hydrogen bond donor with Tyr99 (A) for 95.41% of the time and π-stacking interactions with Tyr100 (A) for 81.75% of the time, in addition to interactions with hydrogen bond acceptors Tyr100 (A) of 98%, which decreases in cluster 2 (to 62.65%) as well as the frequency of the π-stacking interaction with Tyr349 (B) (from 18.24 to 9.03%) to strengthen the interaction with Thr316 (B) (from 1.99 to 37.34%) (Figure A2). 

In BP2, only three types of interaction are present in the three groups (hydrophobic, hydrogen bond donors and hydrogen bond acceptors), Tyr99 (A) being the only hydrogen bond donor interaction with an interaction frequency between 96 and 100% over time in the three clusters (Figure A2). It should be noted that in the interaction frequencies of each cluster, the number of interactions and the type of cation-π influence the stability of the union, as is the case with BP2, which does not present it [48].

The RMSF showed a very similar fluctuation pattern between the free protein and the protein in complex with the ligands, especially with the BP5 compound. However, the RMSF calculation revealed that the complex with the BP2 ligand showed a high fluctuation in some regions according to the RMSD pattern. Additionally, L1-*Tc*TIM and BTS-*Tc*TIM complexes showed a fluctuation of variable proportions.

In the calculation of RMSF, we observe that the high fluctuation in some regions ac-cording to the RMSD pattern may be due to mobile residues in both protein monomers that coincide with the regions of loop 3 (between residues 68 to 79), an adjacent moving α-helix (from residues 120 to 140), and loop 6 (residues 165 to 177), also known as the flexible or catalytic loop, which forms a kind of cap that opens and closes on the active site [15,37,49], being a factor that contributed to the elevation of RMSD values. 

Additionally, the Rg value for all four complexes was slightly lower than that of free *Tc*TIM. Therefore, it can be suggested that these complexes could be relatively more rigid than the free *Tc*TIM. In addition, the similar Rg values in the four complexes suggest that the interactions of the ligands analyzed do not influence the variation of the protein structure (Figure 6).

MMGBSA analysis calculated the binding free energy of the ligand–*Tc*TIM complex [50]. The van der Waals (ΔEvdw) forces form the greatest contribution to the free energy of binding in the three complexes (L1–*Tc*TIM, BP2–*Tc*TIM and BP5–*Tc*TIM), especially with BP5. These kind of forces are more crucial than the electrostatic to determine binding [43,51]. The BP5–*Tc*TIM complex presented the most favorable binding free energy (ΔGb) (−25.68 ± 0.19 Kcal/mol), followed by L1–*Tc*TIM (−24.37 ± 0.21 Kcal/mol).

On the other hand, a low SASA energy (ΔGSA), in the case of the three complexes (L1–*Tc*TIM, BP2–*Tc*TIM and BP5–*Tc*TIM), also indicates that the nonpolar residues are buried in the solvent, which may favor the stability of the complex through the synergistic effects of hydrogen bonding and hydrophobic interactions [50]. Additionally, the BTS–*Tc*TIM complex was analyzed in the same way, where the ΔGSA was the lowest (−2.93 ± 0.01 Kcal/mol), favoring the stability of the complex and the contributions of ΔEvdw as well as the polar contribution (ΔGpolar), which were the ones that formed the highest contribution to ΔGb (−14.12 ± 0.23 Kcal/mol), which compared to the other compounds was the highest, validating the docking score obtained in molecular docking (−6.2 Kcal/mol).

### 3.5. Analysis of Molecular Physicochemical Properties

Compounds BP2 and BP5 were analyzed through the SwissADME website and the ProTox-II server. Neither compound violated any of the physicochemical properties, and, as such, they have drug-like characteristics [52]. The in silico analysis predicted high human intestinal absorption and null permeability of the blood–brain barrier for the two compounds, showing the potential for good oral absorption without causing central nervous system damage. In addition, they presented moderate solubility, an important aspect, since if the compound is not soluble in water, it cannot be absorbed [53].

On the other hand, the compounds showed hepatotoxic inactivity and were substrates of P-glycoprotein, an efflux pump related to drug resistance [54]. This glycoprotein takes the substance that is related to it and incorporates it into its structure to expel it— for example, through the intestine. This is a way in which the cell defends itself against toxic substances [55]. Many compounds can be a substrate of this glycoprotein—for example, it has been observed with antibiotics and anticancer agents [56]. In addition, the CYP450 inhibition prediction results showed that BP2 and BP5 compounds are likely to behave as inhibitors of almost all CYP450 isoforms (except isoform 1A2), potentially causing these isoforms to be unable to metabolize other drugs because they will be inhibited, maybe giving rise to drug–drug interactions [40]; therefore, it is necessary to improve these properties in these kinds of compounds.

### 3.6. Molecular Docking at the HsTIM Interface

To know the selectivity of control ligand L1 and the compounds BP2 and BP5 against *Tc*TIM versus *Hs*TIM, the molecular docking analysis at the *Hs*TIM interface was performed. The results showed that the control ligand L1 exhibited a docking score of −7.2 Kcal/mol at the *Hs*TIM interface. The orientation of the L1 ligand at the *Hs*TIM interface allowed for hydrophobic interactions between the L1 and *Hs*TIM ring systems with amino acid residues Arg17 (B), Asn71 (A), and Leu236 (B). Furthermore, the thiazole of ligand L1 formed a hydrogen bond between the thiazole ring nitrogen and guanidino nitrogen (C(NH_2_)^+3^). Compound BP2 showed a higher affinity, with a docking score of −8.0 Kcal/mol, than the control ligand L1 for the *Hs*TIM interface. Due to its structure formed by a ring system, it allowed it to interact with many residues, such as Gly16 (B), Arg17 (B), Gln19 (B), Ser20 (B), Leu21 (B), Leu24 (B), Asn71 (A), Asp85 (A), and Leu236 (B). The putative position of the BP2 compound at the interface suggests that it may alter the stability of *Hs*TIM monomers. Compound BP5 exhibited low affinity with a docking score of −5.9 Kcal/mol towards *Hs*TIM. Likewise, the interactions observed were poor with respect to compound BP2 and the control ligand L1. Only three hydrogen bonding interactions with Gly16 (B) and Asn71 (A), and one hydrophobic contact with Thr70 (A), were observed. 

Molecular dynamics simulations for the three complexes (L1–*Hs*TIM, BP2–*Hs*TIM, and BP5–*Hs*TIM) and free–*Hs*TIM were also analyzed (Figure 8). The trajectories were first analyzed by RMSD and RMSF analysis to understand the stability and fluctuations of these structures [50]. Subsequently, the Rg was analyzed to observe the compactness of the system, where BP5 was slightly lower than free-*Hs*TIM, suggesting that this complex may be relatively more rigid. In addition, the free energies of binding were calculated by the MMGBSA method from the molecular dynamics trajectories. The ΔGb values for the three complexes (L1–*Hs*TIM, BP2–*Hs*TIM, and BP5–*Hs*TIM) show lower values than the complexes with *Tc*TIM, supporting the docking scores obtained in molecular docking. In general, these results suggested that BP5 showed lower affinity against *Hs*TIM than LI and BP2.

## 4. Materials and Methods

### 4.1. Data Preparation

10 compounds with inhibitory activity against *Tc*TIM were used as control ligands, which were drawn in ChemDraw and saved in SDF format. Afterwards, they were minimized and converted to pdbqt format from OpenBabel.

### 4.2. Molecular Docking

The crystallographic structure of the *Tc*TIM protein in complex with 3-(2-benzothiazolylthio-1-propanesulfonic acid (BTS) was obtained from the Protein Data Bank (PDB) “http://www.pdb.org (accessed on 12 May 2021)” [57] with the PDB ID 1SUX as well as the *Hs*TIM protein with the accession code 4POC. The protein was prepared for docking using the UCSF Chimera 1.14.1 DockPrep tool [58]. Additionally, the prepare_receptor4.py script from MGLTools 1.5.6 was used to add AutoDock atom types and add Gasteiger charges to the protein structure.

The prediction of potential binding sites was performed first using the DoGSiteScorer tool of the Proteins Plus server “https://proteins.plus/ (accessed on 18 May 2021)”. Subsequently, a blind molecular docking was performed. For blind molecular docking, the receptor was defined as rigid and PyRx software was used, which works with AutoDock vina 1.1.2 (vina) [59]. For docking at the binding site, the conformational search space was determined by establishing the coordinates in the center of residues at the interface (X = 29,077, Y = 101,654, and Z = 62,161) using PyRx software. The binding site on *Hs*TIM was determined by overlap between *Tc*TIM (PDB 1SUX) and the *Hs*TIM apoprotein (PDB 4POC) using UCSF Chimera. Then, docking at *Hs*TIM was centered on interface residues, as previously described [56].

### 4.3. Virtual Screening

LBVS using benzimidazole scaffold was performed from the ZINC15 database “https://zinc15.docking.org (accessed on 25 May 2021)” [60]. Subsequently, all structures were downloaded in “CSV” format and the Lipinski’s rule of five was applied using the OpenBabel program. Finally, the structures were prepared for molecular docking on the *Tc*TIM interface using the PyRx program. Once molecular docking at the *Tc*TIM interface was concluded, all compounds were selected based on the docking score of −8.9 Kcal/mol obtained by the L1 ligand (see results section). Subsequently, through the PLIP web service, an interaction profile was generated for each of the docking complexes [61]. With the scikit-learn library and the DataWarrior program “https://openmolecules.org/datawarrior (accessed on 8 June 2021)” [33,62], the compounds were grouped according to the interaction profiles. Finally, two compounds were selected based on cost and availability for evaluation against bloodstream trypomastigotes of *T. cruzi*.

### 4.4. Trypanocidal Assay

CD1 mice, 6 to 8 weeks old, infected with bloodstream trypomastigotes of INC-5 and NINOA strain, were used for the trypanocidal assay. At the peak of parasitemia (2 to 4 weeks), parasitized blood was collected by cardiac puncture using sodium heparin as an anticoagulant. Blood was adjusted to 1 × 10^6^ trypomastigotes/mL. In a 96-well plate, 90 µL of infected blood and 10 µL of benzimidazole derivatives or dilutions of the reference drugs (Bzn and Nfx) were deposited for a final volume of 100 µL per well. Reference drugs were used as positive lysis control, and wells with untreated blood trypomastigotes were used as negative lysis control; the microplates were incubated at 4 °C for 24 h. Motile trypomastigotes were subsequently quantified using the Pizzi–Brener method [63], for which 5 μL of blood was placed on a slide and covered with a 18 × 18 mm coverslip. Motile protozoa were counted in 15 fields at 40× using an optical microscope. The lysis percentage of each treatment was calculated by comparing the viable trypomastigotes with the negative control [64]. The half-maximum lytic concentration (LC_50_) was determined for each compound using the Probit statistical tool. Subsequently, the results were converted to micromolar units. Benzimidazole derivatives (BP2 and BP5) were purchased from the commercial house MolPort and were worked without any additional purification. The assay was performed in triplicate.

### 4.5. Molecular Dynamics Analysis

For analysis of molecular dynamics of the selected compounds, the open-source software suite GROMACS 5.1.2 was used [65]. Protein with access code 1SUX was parameterized in AMBER03 (ff94/ff99 modification from Duan et al., 2003) [66] force field with the GROMACS software suite pdb2gmx. Protein protonation state pH 7 was previously calculated using PROPKA tool implemented in UCSF Chimera. On the other hand, the topology of the compounds was generated with ACPYPE Server “http://webapps.ccpn.ac.uk/acpype (accessed on 14 July 2021)” [67], which is based on the General Amber Force Field (GAFF). The system was a dodecahedron with periodic boundary conditions. In addition to containing the ligand-protein complex, it was filled with TIP3P water molecules and the number of ions (Cl^−^ or Na^+^) necessary to have a neutral charge in the system. Before performing dynamics, the system was energetically minimized by the steepest descent algorithm. Then, two equilibrium steps were performed with restrictions of 1000 kJ/mol/nm^2^ on the movement of protein and ligand heavy atoms. The first stage was at constant pressure, implementing the leapfrog method and the v-rescale thermostat to bring the system from 0 to 300 K. The second stage was carried out at constant temperature, again with the frog jump method, but now with the Berendsen barostat to bring the system from 1 to 2 bar. Both stages achieved a duration of 100 ps. Once the system was balanced, molecular dynamics were performed with a 100 ns trajectory, where long-range interactions and forces were calculated with the Particle-Mesh Ewald (PME) method, establishing the Lennard–Jones and Coulomb contributions at 1.2 nm, balancing the system with samples at 100 ps. Finally, the interactions for each complex in the simulation were obtained with the ProLIF tool (Protein–Ligand Interaction Fingerprints) [68]. The determination of the percentage of the frequencies of interactions was carried out according to the grouping obtained by each complex. The stability of the complexes was determined using the GROMACS software tools. First, the RMSD between the α carbons and the ligand was obtained. Then, the pairwise RMSD matrix was calculated during the 100 ns. It was performed with MDAnalysis [69] to make the RMSD clustering with the metric ward and the Euclidean distance from the python3 SciPy library. RMSF of α-carbons along with 2D structure [70] was performed to understand the effect of the compound on the secondary structure of the TIM surface. Finally, the Rg was calculated to corroborate the maintenance of the three-dimensional compactness of the TIM throughout the simulation. The free energy of binding of the complexes was calculated with the MMPBSA using the g_mmpbsa software [71], retrieving 200 stable frames (between 90 and 100 ns).

### 4.6. Analysis of Molecular Physicochemical Properties

In addition, the compounds BP2 and BP5 were *in silico* analyzed with the SwissADME website “http://www.swissadme.ch (accessed on 22 August 2021)” [72] to determine their pharmacokinetic properties, such as absorption, bioavailability, permeability, blood–brain barrier penetration, metabolism, and excretion, as well as hepatotoxicity via the ProTox-II server “https://tox-new.charite.de/protox_II (accessed on 23 August 2022)” [73].

## 5. Conclusions

In this study, a LBVS using a benzimidazole scaffold and molecular docking at the *Tc*TIM interface allowed obtaining 1604 new benzimidazole derivatives as potential *Tc*TIM inhibitors. The biological evaluation in the in vitro model against the trypomastigote form of *T. cruzi* determined that compounds BP2 and BP5 have trypanocidal activity comparable to reference drugs. Additionally, the molecular dynamics simulation demonstrated that compound BP5 formed the most stable complex with *Tc*TIM during the time analyzed, and van der Waals forces were described as the most important in the binding process. Finally, a low affinity (−5.9 Kcal/mol) of BP5 at the *Hs*TIM interface suggest a selectivity on *Tc*TIM. These results encourage researchers to explore these kinds of compounds to develop new trypanocidal agents. 

## Figures and Tables

**Table 1:** Docking scores and molecular interactions of *Tc*TIM inhibitory compounds used as control ligands at the *Tc*TIM interface.

ID	Compound	Antiparasitic Activity	Enzymatic Activity	Docking Score Kcal/mol	Hydrophobic Interactions	Hydrogen Bonds	π-Stacking Interactions
L1	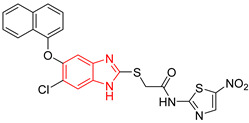	-	% inhibition = 48 at 200 µM [20]	−8.9	Tyr102 (A) Ile69 (B) Arg71 (B) Phe75 (B)	Glu75 (A) Tyr102 (A) Arg71 (B)	Tyr103 (A)
L2	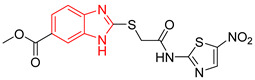	-	% inhibition = 52 at 200 µM [21]	−6.8	Tyr102 (A) Ile69 (B)	Leu101 (A) Tyr102 (A) Tyr103 (A) Arg71 (B)	Tyr103 (A)
L3	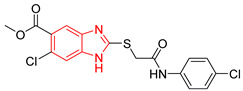	Epimastigotes IC_50_ = 186.23 μM [25]	% inhibition = 65 at 100 µM [21]	−6.9	Tyr102 (A) Ile69 (B)	Tyr103 (A) Arg71 (B)	Tyr103 (A) Tyr103 (B)
L4	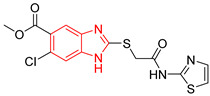	Epimastigotes IC_50_ = 48.91 μM [25]	% inhibition = 59 at 200 µM [25]	−7.3	Tyr102 (A) Ile69 (B)	Tyr103 (A) Arg71 (B)	Tyr103 (A) Tyr103 (B)
L5	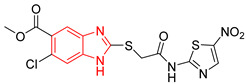	Epimastigotes IC_50_ = 42.51 μM [25]	% inhibition = 69 at 200 µM [25]	−7.3	Tyr102 (A) Ile69 (B)	Tyr103 (A) Arg71 (B)	Tyr103 (A)
L6	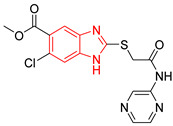	Epimastigotes IC_50_ = 28.67 μM [25]	% de inhibición = 68 at 200 µM [25]	−7.2	Tyr102 (A) Ile69 (B) Phe75 (B)	Tyr103 (A) Tyr103 (B) Gly104 (B)	Tyr103 (A)
L7	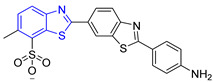	-	IC_50_ = 8.0 µM; % inhibition = 95 at 250 µM [26,27]	−6.9	Tyr102 (A) Ile69 (B) Glu108 (B) Ile109 (B)	Tyr103 (A) Glu112 (B)	Tyr103 (A)
L8	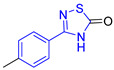	Epimastigotes IC_50_ = 5.8 µM [28]	IC_50_ = 3.5 µM [28]	−6.9	Tyr102 (A) Ile69 (B) Phe75 (B)	Tyr102 (A) Tyr103 (B)	Tyr103 (A)
L9	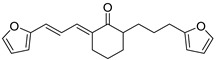	Epimastigotes IC_50_ = 0.6 µM [29]	IC_50_ = 0.086 µM [29]	−7.2	Tyr102 (A) Tyr103 (A) Ile69 (B) Arg71 (B) Phe75 (B) Tyr103 (B) Glu105 (B)	Arg71 (B)	Tyr102 (A)
BTS	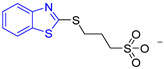	-	semi-maximal inhibition at 35 μM [31]	−6.2	Tyr102 (A) Ile69 (B) Phe75 (B)	Tyr103 (B)	Tyr103 (A)

**Table 2:** Lead compounds from each group obtained by interaction profile analysis of 1604 potential *Tc*TIM inhibitors.

Group	Compounds in the Group	ID Best Composite of Each Group	Docking Score (Kcal/mol)	Structure
1	167	BP1 (ZINC000150134991)	−10.2	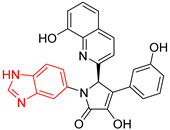
2	135	BP2 (ZINC000040071949)	−10.4	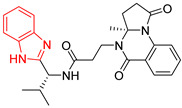
3	206	BP3 (ZINC000030009841)	−10.3	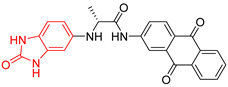
4	171	BP4 (ZINC000150010278)	−10.0	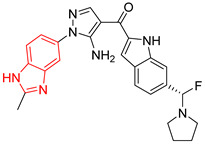
5	142	BP5 (ZINC000040013445)	−10.2	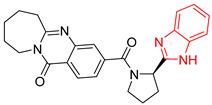
6	103	BP6 (ZINC000140212311)	−10.2	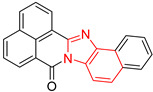
7	181	BP7 (ZINC000040170214)	−10.5	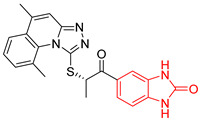
8	162	BP8 (ZINC000040170215)	−10.6	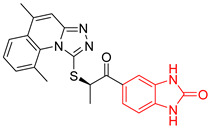
9	191	BP9 (ZINC000170014382)	−10.4	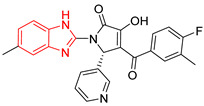
10	146	BP10 (ZINC000170072839)	−10.4	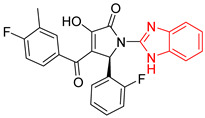

**Table 3:** Trypanocidal activity of benzimidazole derivatives against NINOA and INC-5 strains of *T. cruzi*.

Compound		LC_50_ (µM) *	
		NINOA	INC-5
BP2	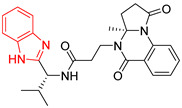	155.86 ± 3.4	226.30 ± 15.4
BP5	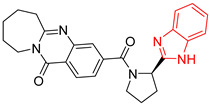	179.55 ± 19.7	179.71 ± 19.0
Nfx		70.41 ± 8.0	139.37 ± 3.0
Bzn		130.72 ± 8.8	191.28 ± 1.8

* The results are means of three experiments.

**Table 4:** Free energy of binding (Kcal/mol) of the Ligand-*Tc*TIM and Ligand-*Hs*TIM complexes.

Complexes	ΔEvdw (kcal/mol)	ΔEele (kcal/mol)	ΔGpolar (kcal/mol)	ΔGSA (kcal/mol)	ΔGb (kcal/mol)
L1-*Tc*TIM	−33.97 ± 0.15	−3.85 ± 0.06	16.99 ± 0.12	−3.89 ± 0.01	−24.73 ± 0.15
BP2-*Tc*TIM	−29.73 ± 0.24	−3.78 ± 0.11	17.77 ± 0.20	−3.28 ± 0.03	−19.03 ± 0.16
BP5-*Tc*TIM	−46.58 ± 0.2	−5.21 ± 0.1	30.61 ± 0.13	−4.49 ± 0.01	−25.68 ± 0.20
BTS-*Tc*TIM	−24.53 ± 0.23	−14.93 ± 0.37	28.28 ± 0.21	−2.93 ± 0.01	−14.12 ± 0.23
L1-*Hs*TIM	−32.19 ± 0.44	−11.71 ± 0.37	34.74 ± 0.67	−3.56 ± 0.03	−12.72 ± 0.25
BP2-*Hs*TIM	−30.68 ± 0.24	−0.09 ± 0.12	14.02 ± 0.2	−2.98 ± 0.02	−19.72 ± 0.21
BP5-*Hs*TIM	−23.04 ± 0.18	−5.22 ± 0.18	15.63 ± 0.83	−2.26 ± 0.02	−14.89 ± 0.84

ΔEvdw, van der Waals contributions; ΔEele, Electrostatic contributions; ΔGpolar, Polar contributions; ΔGSA, SASA contributions, ΔGb; affinity energy.

**Table 5:** Molecular physicochemical properties of BP2 and BP5 using SwissADME website and the ProTox-II server.

Physicochemical Properties														
Compound		*M*_W_ (g/mol) < 500		Nb < 10	Nhba < 10	Nhbd < 5		TPSA (Å^2^) < 140			Log *P* < 5		Log *S* ^1^	
BP2		459.54		7	4	2		98.40			2.70		Moderately soluble	
BP5		427.50		3	4	1		88.88			3.20		Moderately soluble	
**Probability of pharmacokinetic properties**														
Compound	Blood-brain permeability		Human gastrointestinal absorption ^2^		P-glycoprotein substrate	CYP1A2 inhibitor	CYP2C19 inhibitor		CYP2C9 inhibitor	CYP2D6 inhibitor		CYP3A4 inhibitor		Hepatotoxicity
BP2	No		Soluble		Yes	No	Yes		Yes	Yes		Yes		Inactive
BP5	No		Soluble		Yes	No	Yes		Yes	Yes		Yes		Inactive

*M*_W_: molecular weight, Nb: number of rotatable bonds, Nhba: number of hydrogen bond acceptors, Nhbd: number of hydrogen bond donors, TPSA: polar surface area, Log P: partition coefficient, Log S: solubility coefficient. null.

## Data Availability

Not applicable.
